# Where to begin? The best publications for newcomers to ethnopharmacology

**DOI:** 10.3389/fphar.2023.1141502

**Published:** 2023-02-10

**Authors:** Banaz Jalil, Fabien Schultz, Michael Heinrich

**Affiliations:** ^1^ Pharmacognosy and Phytotherapy, UCL School of Pharmacy, London, United Kingdom; ^2^ Department of Agriculture and Food Sciences, Neubrandenburg University of Applied Sciences, Neubrandenburg, Germany; ^3^ Department of Pharmaceutical Sciences and Chinese Medicine Resources, Chinese Medicine Research Center, College of Chinese Medicine, China Medical University, Taichung, Taiwan

**Keywords:** traditional medicine, primary literature, (graduate) education, research methods, ethnopharmacology

## Abstract

Have you ever tried to enter a new field of research or to get a basic overview? Of course, we all have. However, where does one begin when entering a new field of research? This mini-review offers a concise (and certainly not comprehensive) overview on the fast-evolving field of ethnopharmacology. Based on a survey in which researchers provided feedback on the publications they find most relevant in the field and an assessment of what publications have been particularly relevant in the field, this paper offers a review of the 30 best papers and books for newcomers in the field. They cover the relevant areas within ethnopharmacology and give examples from all the core regions where ethnopharmacological research is being conducted. Different and sometimes contrasting approaches and theoretical frameworks are included, as well as publications reviewing important methods. With this, basic knowledge on related fields such as ethnobotany, anthropology, fieldwork methods and pharmacognosy is also incorporated. This paper is an invitation to explore fundamental aspects of the field and to understand the particular challenges faced by researchers newly entering this multi- and transdisciplinary field, and to provide them with examples of particularly stimulating research.

## Introduction

Getting started in any field of research can be a daunting task to a ‘newcomer’. Ethnopharmacology is no exception. Ethnopharmacology studies ‘traditional medicines’, i.e., plants, fungi and other natural substances used as medicines. It is made more complicated by its multi- and transdisciplinary nature, covering such diverse fields like pharmacology, botany, pharmacognosy, analytical and natural product chemistry, anthropology/sociology, biomedical research, toxicology, clinical research, as well as history, environmental science, various areas of biology, climate research and numerous others. The number of publications in the field is impressive. For example, on the Web of Science, as seen in the changes over 10-year periods in annual publications, the keyword ‘traditional medicine’ shows 28532 (2022), 9026 (2012), 2375 (2002), 844 (1992), 49 (1982) and 6 (1972) publications. These numbers show that the science of ethnopharmacology today continues to be a thriving and fast-developing field of research. If one focuses specifically on scientific approaches labelled ‘ethnopharmacology’, for 2022, the Web of Science lists 895 publications, again with an enormous increase over the last 55 years with the specific keyword ‘ethnopharmacology’: 720 (2012), 225 (2002) 62 (1992), 48 (1982) and 0 (1972) publications. With such a large number of publications annually, one can easily get lost in the field.

So, where to begin? In several fields of research, mini-reviews have been published, presenting the most relevant publications (mostly academic papers but also some books), offering a basis for understanding this specific field of research. Examples include pharmacoepidemiology and medicinal chemistry (Giustiniano et al., 2021; Pottegård et al., 2022). Other obvious starting points are websites and digital resources, which however, may not be maintained well or may not have been assessed scientifically. Therefore, here we focus on peer-reviewed publications which are accessible widely.

Ethnopharmacology is – in its initial scope and focus a ‘child of the 1960’s in the global West, i.e., North America and Western Europe. The first use of the term ‘ethnopharmacology’ in writing is linked to a symposium ‘Ethnopharmacological Search for Psychoactive Drugs’ in 1967 and the resulting publications (Efron et al., 1967) and (Holmstedt, 1967). This is much later than, for example, the terms ‘ethnobotany’, which was first used in writing in 1896 describing the study of human’s plant use (Fewkes, 1896; Harshberger, 1896), or ‘pharmacognosy’, which was first used by Schmidt in 1811 (Heinrich, 2015; Balick and Cox, 2020). Related concepts are medical and economic botany, two fields of research at the interface of botany/medicine and botany/useful plants more generally, respectively, as exemplified by the classical book by Lewis and Elvin-Lewis (2003) or Lindley (1849). However, there is far less of a focus on pharmacological and anthropological aspects.

Numerous studies exemplify the (most notably European) interest in exotic medicines resulting from the colonial expansion of European empires and the United States (Heinrich, 2013). Of course, natural products were the only source of medicines until very late in the 19th century CE. While it initially incorporated pharmacology and the local/traditional use of plants and fungi, the focus was exclusively on hallucinogens and other CNS active mind-altering drugs and medicines. As such, it is in the tradition of researchers like most notably Louis Levin (1850–1929), who both covered the pharmacology, chemistry, and the traditional use of hallucinogens, as well as the studies of numerous psychiatrists (natural product), chemists, medical doctors and pharmacists. Within the following decades, the focus of the field broadened to cover a wide range of studies on medicinal plants, their local/traditional uses, their occurrence, conservation, and distribution, their quality assessment in terms of active ingredients and identity, and most importantly, their pharmacology/toxicology (Heinrich, 2015).

In such an overview, the aim is to present publications which are widely accessible, written in such a way that they are understandable to a wide range of researchers, representative of the field both present and past, and especially addressing newcomers to the field of ethnopharmacology.

## Approach and methods

Initially, emails were sent out to all editors and associate editors at the Frontiers in Pharmacology section Ethnopharmacology to invite nominations (but not limited to publications in specific journals) of the most important papers or books in the field of ethnopharmacology. Next, an online survey was also conducted to invite global nominations of important papers or books and those with the greatest influence. Participants were asked explicitly to also include ‘overlooked’ papers or books which are considered important but which should receive more attention since they are fundamental contributions to the field. The online survey comprised one main section with three questions covering (1) full bibliographic details of the nominated paper or book; (2) a short justification of why this paper or book is so essential; and (3) a summary of the main findings of the nominated paper or book. The survey was designed to be short and take 3–5 min to complete (for online survey questions, see Supplementary Table S1). Between June – October 2022, the survey link was distributed *via* a range of scientific society websites, official social media platforms (e.g., LinkedIn, Instagram, Facebook and Twitter), and through personal networks of academics (i.e., using the snowballing approach). All nominations were then solicited between November and December 2022. Our call received considerable attention from ethnopharmacologists all over the world. During data analysis (of responses received), duplicates were merged. Each of the assessors, the authors of the present work, then discussed the remaining nominations focussing on those selected papers or books with three or more votes. This was followed by rounds of adjustments to prioritise between partially overlapping nominations, resulting in a final selection of 30 publications in ethnopharmacology.

Core inclusion criteria were: All papers had to (a) be in English, published as an article (both original studies and reviews) or as a book; (b) be of general interest to the field because of their approach, topic or methodology; (c) be published after the term ‘ethnopharmacology’ was coined; and (d) there was no direct overlap with other studies, i.e., a similar theme. Papers and books listed more than three times in the survey were all included. Papers with a lower frequency of mention were included on a selective basis. In general, preference was given to more widely cited publications.

## Results and discussion

In the initial round, we received 185 nominations. After the merging of duplicates, 110 nominations remained. 15 books and papers suggested three or more times were included in the list of the 30 most relevant publications. The authors then selected 15 additional publications to ascertain a global coverage that the various approaches in ethnopharmacology are represented. This selection is a mix of publications that have had a high impact (e.g., highly cited) and a selection of classical publications of fundamental contribution to the field covering the period since 1967 (Table 1).

**TABLE 1:** Selected most relevant publications in the field of ethnopharmacology (n = 30).

Author (year)	Tittle	Journal/Publisher
Anwar et al. (2007)	*Moringa oleifera*: a food plant with multiple medicinal uses	Phytotherapy Research
Cos et al. (2006)	Anti-infective potential of natural products: How to develop a stronger *in vitro* ‘proof-of-concept’	Journal of Ethnopharmacology
Efron et al. (1967) (reprint: Prance et al. (2018)	Ethnopharmacologic Search for Psychoactive Drugs	Synergetic Press
Ekor. (2014)	The growing use of herbal medicines: issues relating to adverse reactions and challenges in monitoring safety	Frontiers in Pharmacology, Sect. Ethnopharmacolgy
Farnsworth et al. (1985)	Medicinal plants in therapy	Bulletin of the World Health Organization
Gertsch. (2009)	How scientific is the science in ethnopharmacology? Historical perspectives and epistemological problems	Journal of Ethnopharmacology
Grover et al. (2002)	Medicinal plants of India with anti-diabetic potential	Journal of Ethnopharmacology
Gurib-Fakim. (2006)	Medicinal plants: traditions of yesterday and drugs of tomorrow	Molecular Aspects of Medicine
Heinrich and Jäger. (2015)	Ethnopharmacology	John Wiley & Sons
Heinrich et al. (2018a)	Best practice in research: consensus statement on ethnopharmacological field studies–ConSEFS	Journal of Ethnopharmacology
Heinrich et al. (2020)	Best practice in research–Overcoming common challenges in phytopharmacological research	Journal of Ethnopharmacology
Heinrich et al. (2022)	Best Practice in the chemical characterisation of extracts used in pharmacological and toxicological research—The ConPhyMP—Guidelines	Frontiers in Pharmacology, Sect. Ethnopharmacolgy
Hu et al. (2022)	The Adverse Reactions of Lianhua Qingwen Capsule/Granule Compared with Conventional Drug in Clinical Application: A Meta-Analysis	Frontiers in Pharmacology, Sect. Ethnopharmacolgy
Jiang et al. (2020)	An “essential herbal medicine”—Licorice: A review of phytochemicals and its effects in combination preparations	Journal of Ethnopharmacology
Klayman. (1985)	Qinghaosu (artemisinin): an antimalarial drug from China	Science
Lam et al. (2015)	PHY906 (KD018), an adjuvant based on a 1800-year-old Chinese medicine, enhanced the anti-tumor activity of Sorafenib by changing the tumor microenvironment	Scientific Reports
Lansky and Newman. (2007)	*Punica granatum* (pomegranate) and its potential for prevention and treatment of inflammation and cancer	Journal of Ethnopharmacology
Leonti and Casu. (2013)	Traditional medicines and globalization: current and future perspectives in ethnopharmacology	Frontiers in Pharmacology, Sect. Ethnopharmacolgy
Neuwinger. (1996)	African Ethnobotany: Poisons and Drugs—Chemistry, Pharmacology, Toxicology	Chapman & Hall
Ortiz De Montellano. (1975)	Empirical Aztec Medicine: Aztec medicinal plants seem to be effective if they are judged by Aztec standards	Science
Pawar et al. (2021)	Oral curcumin with piperine as adjuvant therapy for the treatment of COVID-19: a randomized clinical trial	Frontiers in Pharmacology, Sect. Ethnopharmacolgy
Reyes-García. (2010)	The relevance of traditional knowledge systems for ethnopharmacological research: theoretical and methodological contributions	Journal of Ethnobiology and Ethnomedicine
Rivera et al. (2014)	What is in a name? The need for accurate scientific nomenclature for plants	Journal of Ethnopharmacology
Schultes and Raffauf. (1990)	The Healing Forest: Medicinal and Toxic Plants of the Northwest Amazonia	Dioscorides Press
Shikov et al. (2014)	Medicinal plants of the Russian Pharmacopoeia; their history and applications	Journal of Ethnopharmacology
Soejarto et al. (2005)	Ethnobotany/ethnopharmacology and mass bioprospecting: Issues on intellectual property and benefit-sharing	Journal of Ethnopharmacology
Tan et al. (2016)	*Gynura procumbens*: An Overview of the Biological Activities	Frontiers in Pharmacology, sect. Ethnopharmacology
Wasser. (2002)	Medicinal mushrooms as a source of antitumor and immunomodulating polysaccharides	Applied Microbiology and Biotechnology
Van Wyk et al. (2009)	Medicinal Plants of South Africa	Briza Publications
Wynberg. (2004)	Rhetoric, Realism and Benefit-Sharing: Use of traditional knowledge of Hoodia species in the development of an appetite suppressant	The Journal of World Intellectual Property

There can be no ‘first’ ever publication in the field since human’s reflections and experimentation with medicinal plants are intrinsic elements of human cultural, social and economic development. There are, however, some seminal studies which demonstrate the wealth of traditions in writing, and these are best known for European and Southeast Asian traditions but also for parts of the Americas (De La Cruz, 1991) [orig. 1552]; (Fuchs, 2022); [orig. 1543] and the many similar works published during this century representing the European tradition first exemplified in the work of Pedanius Dioscorides (ca. 40–90 C.E.) and its re-editions/re-interpretations. Shizhen Li’s *Bencao Gangmu* (*Compendium of Materia Medica*) [orig. completed 1578], first printed in 1596, first English translation in 2003 (Li, 2003), with a new translation being published in numerous volumes since 2021 (Li, 2021), is a memontous study capturing the diverse Chinese traditions. The Canon of Medicine (*al-Qanun fi al-Tibb*) by Ibn Sina (also known as Avicenna) (Avicenna, 2015) [orig. 1052] is among the most influential earlier medical knowledge books, a synthesis of Roman medicine, Greek philosophy and Islamic teaching, supplemented with Ibn Sina’s observations. In particular, volume two on the Materia Medica is of importance, where he lists 800 plants, animal substances and minerals used as medical treatment.

The 19th century saw a flourishing of studies focusing on botanical drugs and their potential based on the colonial expansion and subsequent exploration combined with the fast development of pharmacological studies of these ‘new’ resources (Bernard, 1864; Hartwich, 1897). In this context, one must highlight the work of R. Schultes, who, starting in 1941, spent years in the Columbian Amazon, tasked with finding new sources of rubber and, at the same time, studying the medicinal plants of the rainforest (Schultes and Raffauf, 1990). Watt and Breyer-Brandwijk (1962) were selected to represent the earlier descriptive studies on African medicinal plants. The earliest works defining the original scope of the field as we know it today are studies by Efron et al. (1967) (including later reprints like Prance et al. (2018)), Holmstedt (1967), and the study by Ortiz De Montellano. (1975), assessing the pharmacopoeia of the historic Aztecs based on the criteria as the Aztecs had applied.

In the 1980s, two core development were the increasing importance given to medicinal plants by the WHO (Farnsworth et al., 1985) and the discovery and development of qinghaosu from *Artemisia annua* L. (Klayman, 1985), which has become one of the main therapeutic options for treating malaria, most notably in African and Asian countries and famously led to awarding a Nobel Price in Physiology or Medicine to YouYou Tu in 2015 (Tu, 2004; Tu, 2011; Su and Miller, 2015; Tu, 2017). These studies also exemplify the emerging expansion of the field’s focus into other areas of health and healthcare (beyond psychoactives) (Table 1).

Two more descriptive studies represent the 1990s – Schultes and Raffauf (1990) and Neuwinger (1996). R.E Schultes’ seminal studies on medicinal plants from the American rainforest and the associated chemical and pharmacological assessment by Raffauf resulted in a book with the capturing title ‘The Healing Forest’, which was one of the starting points of a renewed interest in exploring the pharmacology of ‘exotic’ plants. Neuwinger (1996) is a monumental review of the chemistry, pharmacology, and toxicology of African poisons, which are also used as medicines.

The 2000s see a massive flourishing of studies (Grover et al., 2002; Wasser, 2002; Rios and Recio, 2005; Soejarto et al., 2005; Cos et al., 2006; Gurib-Fakim, 2006; Lansky and Newman, 2007; Gertsch, 2009; Van Wyk et al., 2009). Several of these represent reviews either of medically and economic plant species like Lansky and Newman (2007) covering *Punica granatum* (See also Anwar et al. (2007) on *Moringa oleifera* and a decade later Tan et al. (2016) on, *Gynura procumbens*) indicating an interest in the larger commercial development of such health food or medicinal plants. Another core area are emerging areas of pharmacological focus, most notably diabetes, Grover et al. (2002). Soejarto et al. (2005) is a core text for understanding the policy and legal changes resulting from the Convention of Biological Diversity of 1992 and the later Nagoya Protocol (2010). The 1990s and 2010 also saw a sharpening of the critique relating to colonialistic, exploitative and euro-/US-centric uses of biodiversity, including the use of medicinal plants (Wynberg, 2004), a challenge which goes well beyond ethnopharmacology. Gurib-Fakim (2006) offers an important perspective from a country with an emerging economy, while Reyes-García (2010) puts traditionality at the centre of focus again. Others highlight an emerging trend to develop best practice guidelines, for example, Cos et al. (2006) defining guidelines for assessing the anti-infective effects of plant extracts and isolated metabolites (cf. also Rios and Recio (2005) or the wider scientific requirements for meaningful research Gertsch (2009)). Wasser (2002) is included as an example of assessing medicinal mushrooms from an ethnopharmacological perspective. Others continue the more descriptive tradition of the field (Van Wyk et al., 2009). Since at least the 1990‘s there has also been increasing criticism of colonial and exploitative approaches in the field (Table 1).

At least since the 2000‘s there has been a very strong shift of focus and a rise of research on medicinal plants used in numerous Asian traditions, most importantly in the People’s Republic of China, e.g., Lam et al. (2015) and Jiang et al. (2020). A series of best practice studies were published, some focusing on the requirements of a single journal (Weckerle et al., 2018) with the authors using an approach which is driven by experts’ feedback and consensus processes (Rivera et al., 2014; Heinrich et al., 2018a; Heinrich et al., 2020; Heinrich et al., 2022) with not all included in the core list (Table 1). With these studies and taking account of the multidisciplinary nature of the field, a common basis is provided, which enables a reporting of research in the tradition of the numerous consensus statements like the Consort statement for reporting clinical trials (Schulz et al., 2010). Clinical research is also gaining more and more attention (Pawar et al. (2021). The first textbook specific to the entire field was published in 2015 (Heinrich and Jäger, 2015), and common themes emerged further – the globalisation of local and traditional medicines (Leonti and Casu, 2013), biodiversity research, conservation (Kew, 2020; Laird et al., 2020) and climate change (Applequist et al., 2020). The regulations provided by the Nagoya Protocol and the Convention of Biological Diversity and its implementation remain important aspects of the work of ethnopharmacologists that often view themselves as advocates of the respective local communities they collaborate with (Soejarto et al., 2005; Herman, 2018; Schultz et al., 2021).

Safety and, specifically, drug interactions remain an area which has received comparatively little attention, despite of calls to focus more on it (Ekor, 2014), including emerging concerns about herb-drug interactions using clinical approaches (Hu et al., 2022) and there is a focus on using pharmacopoeias as a basis for ethnopharmacological research (Shikov et al., 2014).

Textbooks providing fundamental knowledge on neighbouring fields involved in ethnopharmacological research were not included in the list of the 30 most relevant publications. Yet, these publications can be of great help to newcomers entering the field of ethnopharmacology. Some excellent examples focusing on (a) drug discovery and pharmacognosy (Badal and Delgoda, 2017; Samuelsson and Bohlin, 2017; Heinrich et al., 2018b; Newman and Cragg, 2020); (b) ethnobiology/ethnomedicine (Heinrich et al., 1998); (c) economic botany/ethnobotany (Cunningham, 2001; Martin, 2019; Balick and Cox, 2020); (d) the ethnopharmacology of food (Etkin, 2006); (e) anthropology/sociology (e.g. on research methods: Bernard (2017); (f) botany (e.g. on botanical collections: Bridson and Forman (2010) and (g) phytochemistry (Kinghorn, 2001; Phillipson, 2001) are given. A relevant field manual providing selected guidelines useful to newcomers to ethnopharmacological and ethnobotanical fieldwork is Alexiades and Sheldon (1996).

## Conclusion

This mini-review is intended to enable newcomers to the field to understand the wide conceptual basis and the diverse general methodologies and approaches in this large field of research. As such, it is an orientation driven by feedback received from the scientific community. There remain some limitations of the approach, for example, due to the way the questionnaire was disseminated (esp., LinkedIn and Twitter), limited feedback was received from some countries, most notably the PR China. We, therefore, searched core journals for widely cited publications with a broad scope, but clearly, this is somewhat based on personal choice. Since this review is intended to be a starting point to understand the field, this is not a major limitation. We also acknowledge the numerous gaps, but by highlighting the top 30 publications, we can only offer a starting point for further exploring the field.

There is an element of personal choice and a focus on best practices in research. We have to refer colleagues to specialist publications for specific methods like *in vitro* or *in vivo* tools for assessing pharmacological, toxicological and microbiological activities or the study of clinical aspects. It was our aim to represent examples of the diversity of views within the field, and as such, we also showcase some of the important debates in the field.

The authors of this paper consider a strong social science element (esp. anthropology) to be a core element of ethnopharmacology. The wider context of biodiversity research, drug discovery, natural product research and other core fields of research, including the policies relevant to ethnopharmacological research (like access and benefit sharing and sustainable sourcing, the impact of climate change on the use of traditional medicines), have only been touched upon briefly. For modern studies, we limited ourselves to publications in English, and it is essential to remember that a field like ethnopharmacology is driven by national, regional and local developments with a rich body of literature in many languages.

We hope that this paper will serve as an orientation and as a basis for giving and developing postgraduate and undergraduate courses. However, in the end, readers should and must make their own choices and select what is most relevant to them and their specific interests. The most important benefit of this overview is that it enables readers to delve into aspects of ethnopharmacology they have not yet explored.
