# Supplementary material for: Where to begin? The best publications for newcomers to ethnopharmacology
Source: Front Pharmacol. 2023 Feb 10;14:1141502. doi: 10.3389/fphar.2023.1141502 (PMC9950406; doi:10.3389/fphar.2023.1141502)
Supplement: Supplementary file 1 [file Table1.pdf]

Core papers in Ethnopharmacology

Start of Block: Default Question Block

Q1
Core papers in Ethnopharmacology Initiative

Have you ever asked yourself how to get some core basic information on a field of research you are getting active in? We are a group of ethnopharmacologists putting together a list of core papers you should read if you are new to the field! Would you like to nominate? What papers or books should we consider?

In this context, important papers or books could be those which have had the greatest influence, but also papers or books which you consider important but which should receive more attention since they are fundamental contributions in the field, which have, however, not been taken into consideration enough (or which are simply older).

We would like to invite you to nominate ONE such paper or book. If you would like to contribute, please provide below the
(1) full bibliographic details;
(2) a short justification of why this paper or book is so essential (optional); and
(3) a summary of the main findings of the paper or book proposed (optional).

We will then collate them here in the UK and, based on the feedback, decide on how we will take it forward, presumably in the form of an outreach article. If you want to be kept up to date, please include your name and email address. Papers from your own group or yourself (i.e., self-nominations) are excluded.

Your contribution is of course, voluntary, no personal identifiable data will be collected aside from your name, and email address and all personal data we collect will be confidential. Your nominated paper or book will NOT be associated with your name or email address.

Thank you very much for your contribution

Q2 **What do you consider to be the best paper or book?**

- [ ] Please provide the full bibliographic details (authors (year) title, journal/source, volume and pages, DOI) of the paper (or book) proposed
____

- [ ] Please provide a short justification for the paper (or book) proposed (optional)
____

- [ ] Please provide a summary of the main findings of the paper (or book) proposed (optional) ____

Q3 **If you would like to be kept up to date about this initiative, please provide your name and email address (optional)**

- [ ] Your name (optional)
____

- [ ] Email address (optional)
____

End of Block: Default Question Block
